# Sound-Absorption Coefficient of Bark-Based Insulation Panels

**DOI:** 10.3390/polym12051012

**Published:** 2020-04-29

**Authors:** Eugenia Mariana Tudor, Anna Dettendorfer, Günther Kain, Marius Catalin Barbu, Roman Réh, Ľuboš Krišťák

**Affiliations:** 1 Forest Products Technology and Timber Construction Department, Salzburg University of Applied Sciences, Markt 136a, 5431 Kuchl, Austria; 2 Faculty of Wood Engineering, Transilvania University of Brasov, Bld. Eroilor nr.29, 500036 Brasov, Romania; 3 Steelcase, Brienner Str. 42, 80333 München, Germany; 4 Faculty of Wood Sciences and Technology, Technical University in Zvolen, T. G. Masaryka 24, SK-960 01 Zvolen, Slovakia

**Keywords:** spruce and larch bark, sound absorption coefficient, impedance tube, biomass, up-cycling

## Abstract

The objective of this study was to investigate the sound absorption coefficient of bark-based insulation panels made of softwood barks Spruce (*Picea abies* (L.) H. Karst.) and Larch (*Larix decidua* Mill.) by means of impedance tube, with a frequency range between 125 and 4000 Hz. The highest efficiency of sound absorption was recorded for spruce bark-based insulation boards bonded with urea-formaldehyde resin, at a level of 1000 and 2000 Hz. The potential of noise reduction of larch bark-based panels glued with tannin-based adhesive covers the same frequency interval. The experimental results show that softwood bark, an underrated material, can substitute expensive materials that involve more grey energy in sound insulation applications. Compared with wood-based composites, the engineered spruce bark (with coarse-grained and fine-grained particles) can absorb the sound even better than MDF, particleboard or OSB. Therefore, the sound absorption coefficient values strengthen the application of insulation panels based on tree bark as structural elements for the noise reduction in residential buildings, and concurrently they open the new ways for a deeper research in this field.

## 1. Introduction

Noise control is an important issue in modern life. A lot of factors contribute to its increase, e.g., population growth, expansion of the urban centers, densification of the housing sector, correlated to the number of vehicles, the development of automatic machines in industrial companies and devices [1,2,3,4].

Noise pollution is the second most important environmental factor in Europe, North America and South-East Asia, contributing to different diseases after air pollution. People of all age groups are becoming more vulnerable to mental stress, heart diseases, sleep disturbance, tinnitus, learning disabilities etc. [5,6,7]. The range of frequencies for the human voice and musical sound is mostly from 125 to 3000 Hz [8,9]. The human audible frequency range extends up to 15 kHz for most persons, and can reach 20 kHz for children and young people [10]. The sound absorption coefficient gives information about the acoustical effectiveness of a material and is defined as the fraction of the energy of incident sound waves absorbed by the material [1,4]. The values of the sound absorption coefficient are between 0 (no absorption) and 1 (complete absorption, e.g., acoustical walls in recording studios) [11,12,13]. Sound insulation (expressed as the transmission loss factor) and absorption are two different properties. Materials that are effective as sound insulators are mostly not useful as sound absorbers and vice versa. Parameters that influence values of these sound insulation and absorption include density, porosity and material thickness. Thicker, denser, and heavier material have higher transmission loss factor values, and porous materials are more effective at sound absorption [14,15,16,17].

Noise control in buildings is ensured through insulation from external sound sources and absorption of sound generated within a space by blocking the transmission of sound from a room to another [18]. 

Non-woven materials have been analyzed as sound absorption materials by various researchers who studied cotton [19], cellulose [20] and needle [21]. Rwawiire et al. [22] investigated the sound insulation characteristics of non-woven fabric from the inner bark of three Ficus species. The measurements revealed that the sound absorption of the bark cloths have higher sound absorption properties at higher frequencies, with an improved absorption coefficient when increasing the bark cloth fiber layers [22]. The planks of birch bark were used for a hundred years as sound absorbers under turf roofs in Sweden, serving also as waterproof membrane [23]. Natural materials such as bark, jute, flax, kenaf, hemp, coir fiber, wood, wool, coconut, straw, cane and corn husk can be designed as thermal and sound absorbers with the advantage of availability [24,25,26,27], sustainability [28,29,30,31,32,33,34,35] and biodegrability [36,37,38,39,40,41,42]. 

This paper presents some aspects about sound absorption properties of tree bark insulation panels made of larch (*Larix decidua* Mill.) and spruce (*Picea abies* (*L*.) H.Karst.), with different particle sizes. The sound absorption coefficient of these panels was compared with wood-based composites from a previous research conducted by Smardzewski et al. [9]. This is an example of the upcycling application of woody biomass as resource for sound and thermal insulation boards that can diminish the noise level in a building. In the context of the scarcity of raw materials, the cascading use of wood and forest residues plays an important role, and should be weighed as a basic concept within the circular economy [43,44]. Bio-economy can be considered environmentally beneficial only if the bio-based resources are managed sustainably [45].

## 2. Materials and Methods

The spruce and larch bark were collected in a local sawmill in Salzburg County, Austria. The bark planks were ground by means of a 4-shaft shredder RS40 at Untha Co. (Kuchl, Austria), with a mesh of 30 mm. Subsequently, the bark particles were dried at 60 °C and 200 to 250 mbar in a vacuum kiln dryer Brunner-Hildebrand High VAC-S, HV-S1 from 65% to 9.0% moisture content. 

The material was repeatedly screened according to EN 15149-1:2011 [46] with a sieve shaker Retsch AS 200, to obtain particles in a size spectrum of 8–13 mm and 10–30 mm.

Four types of bark-based insulation boards were manufactured (Table 1). The spruce bark was bonded with 10% urea formaldehyde type Prefere 10F102 (MetaDynea Austria, Krems, Austria). The larck bark from Graggaber sawmill, Unternberg, Austria was glued with 10% tannin-based adhesive. The formulation included Mimosa tannin extract powder (*Acacia mearnsii*) from Phenotan, Tanac, Brasil, hexa-methylenetetramine (hexamine) from Merck Schuchardt, Hohenbrunn, Germany (C99 %) and sodium hydroxide solution (C32 %) from Carl Roth, Karlsruhe, Germany. A total of 50% tannin extract powder and 50% water were stirred with a mechanical mixer at 700 and 1500 rpm. A total of 10% of hexamine was added and sodium hydroxide was used to adjust the pH value to 9. The boards were pressed at 180 °C for five minutes with a press factor of 24 s/mm [47,48]. 

100 mm diameter samples were cut from bark-based insulation boards manufactured in the laboratories of Salzburg University of Applied Sciences. The thermal conductivity, mechanical properties, microstructures and volatile organic compounds (VOC) emissions of these panels were analyzed in publications by Kain et al. [48,49,50,51,52].

The samples were prepared from different areas of the boards and cut according to EN 326- 1:1994 [53] and ISO16999 [54]. The average moisture content of the samples was 8–9%, according to EN 322:2005 [55]. 

### Acoustical Measurements in Impedance Tube

**Figure 1:**
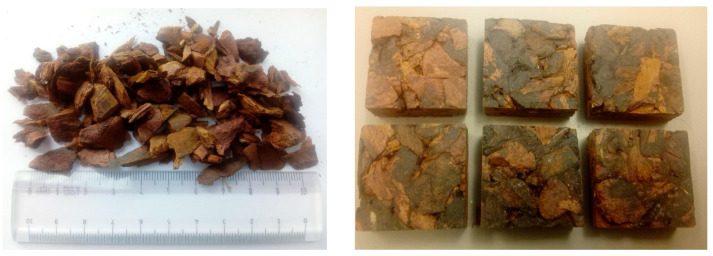
Larch bark particles (8–13 mm) (left) and bark based composite specimens (right) for acoustic measurements.

The acoustical properties of bark-based insulation panels (Figure 1) were measured with an impedance tube system at Krämer & Stegmaier, Berlin, Germany, according to EN ISO 10534-2:2001 (transfer function method) [56]. The system consists of two different sized pipes, which are optimized for high and low frequencies. This covers a frequency range from 50 to 5000 Hz. In contrast to measurements in the reverberation chamber, only small material samples are required for the impedance tube (30 or 150 mm edge length) [57].

The test specimen was introduced in a stationary acoustic field generated by a speaker under normal incidence. The absorption coefficient and the impedance were determined using the transfer function between two microphones. By evaluating the incident and reflected sound energy, the sound absorption capacity of the material was determined [58]. The precision of results depends on the design specifications of the impedance tube. These are the diameter of the tube, the distance of the microphones from the samples and the distance between the microphones [59].

The share of sound absorbed by the bark-based insulation samples was calculated using Equations (1) and (2):

(1) $$\alpha =\frac{I_{i}}{I_{r}}\equiv \frac{{\left|P_{i}\right|}^{2}-{\left|p_{r}\right|}^{2}}{{\left|p_{i}\right|}^{2}}=1-{\left[\frac{n-1}{n+1}\right]}^{2}=\frac{4n}{{\left(1+n\right)}^{2}}$$

(2) $$n=\frac{p_{max}}{p_{min}}$$

where

α is he sound absorption coefficient

$I_{i}$ and $I_{r}$ are intensities of incident and reflected waves

$p_{i}$ and $p_{r}$ are the pressures of incident and reflected waves

n is the standing wave ratio (the ratio of the maximum $p_{max}$ to minimum $p_{min}$ pressure of the sound wave) [60] cited by [61].

## 3. Results and Discussion

The measurements were carried out with and without wall clearance. The assessments with wall clearance are relevant for products such as multi-layered acoustic panels with cavities.

The acoustical properties of materials can be determined by means of impedance tube. In order to calculate the sound absorption capacity of the larch bark samples, a sound wave was emitted in the direction of the test specimen; and then the reflected sound energy was determined.

**Figure 2:**
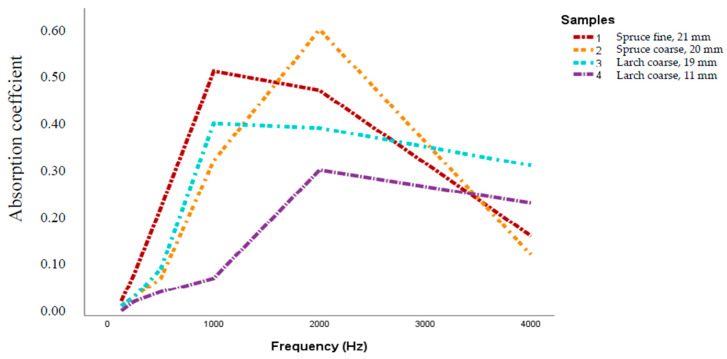
Sound absorption coefficient for spruce and larch bark insulation boards, measured without wall clearance.

Figure 2 shows how much sound was absorbed by the individual samples at a frequency ranging from 125 to 4000 Hz, without wall clearance. 

The thickness of the board plays an important role for the sound absorption coefficient (α), considering here the samples with 19, 20 and 21 mm, compared with the one with 11 mm. The contribution of bark insulation boards to a better sound absorption can be observed in the frequency range of 1000–2000 Hz [9,42,62] when α increases significantly and has a peak at 2000 Hz, at α = 0.61 for the 21 mm sample of spruce board at a density level of 414 kg/m³, compared to α = 0.31 for the 11 mm larch bark sample with a density of 693 kg/m³. Two other peaks were recorded at 1000 Hz for the spruce bark board (α = 0.52) made with fine particles (500 kg/m³) and for the larch bark thick board (α = 0.41) with coarse particles (571 kg/m³). After 2000 Hz α decreases for all testing specimens, meaning that the boards are able to absorb noise at a level of absorption coefficient smaller than 0.62. This experiment shows that the sound absorbing properties of bark based insulating materials can be enhanced by reducing density and increasing thickness. These results are in compliance with Arenas and Crocker [16] and McMullan [17]. Based on these studies, materials that possess a high value of sound absorption are usually porous materials. Sound absorption behavior observed for low-density particleboard showed that this board had higher porosity compared to medium-density particleboard.

**Figure 3:**
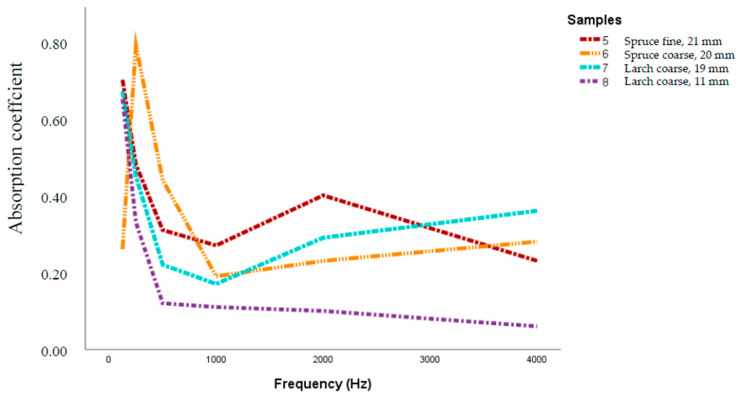
Sound absorption coefficient for spruce and larch bark insulation boards, measured with wall clearance.

In case of using wall clearance (Figure 3), the sound absorption effect is high at low frequencies, ranging from 125 Hz (very close values, from 0.65 to 0.7). The peak is reached by the 20 mm thick sample made of spruce, with a level of α = 0.79 at 250 Hz. After this frequency, the absorption coefficient decreases abrupt until 1000 Hz, with a short increment up to 0.44 for spruce bark sample with fine-grained particle size (8–13 mm). After that α ranges from 0.36 to 0.06 at 4000 Hz. The lowest value means almost no sound absorption.

**Figure 4:**
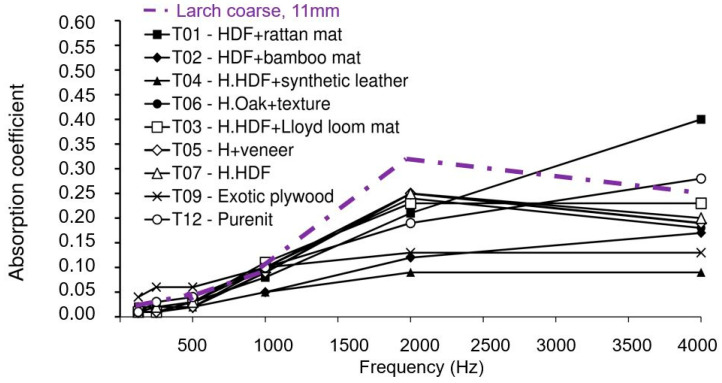
Dependence of the sound absorption coefficient on frequency for wood-based composites with a thickness 7.9 < x < 11 mm (after Smardzewski et al. [9]).

Smardzewski et al. [9] analyzed the sound absorption of 17 different wood-based materials. For a thickness of 7.9 < x < 11 mm, the highest absorption coefficients were recorded between 1000 Hz and 2000 Hz for honeycomb (T07, for a 10 mm thickness: α = 0.10 (1000 Hz), α = 0.25 (2000 Hz)), honeycomb + veneer (T05, for a 9.8 mm thickness: α = 0.09 (1000 Hz), α = 0.25 (2000 Hz)), honeycomb + oak + texture (T06, for a 10.5 thickness: α = 0.10 (1000 Hz), α = 0.24 (2000 Hz)) and honeycomb + Lloyd loom mat (T03, for a 10.2 mm thickness: α = 0.11 (1000 Hz), α = 0.23 (2000 Hz)), compared to α = 0.08 and α = 0.31 at 1000, respectively 2000 Hz for the 11 mm larch bark board, that performed better for this interval of frequencies (Figure 4).

**Figure 5:**
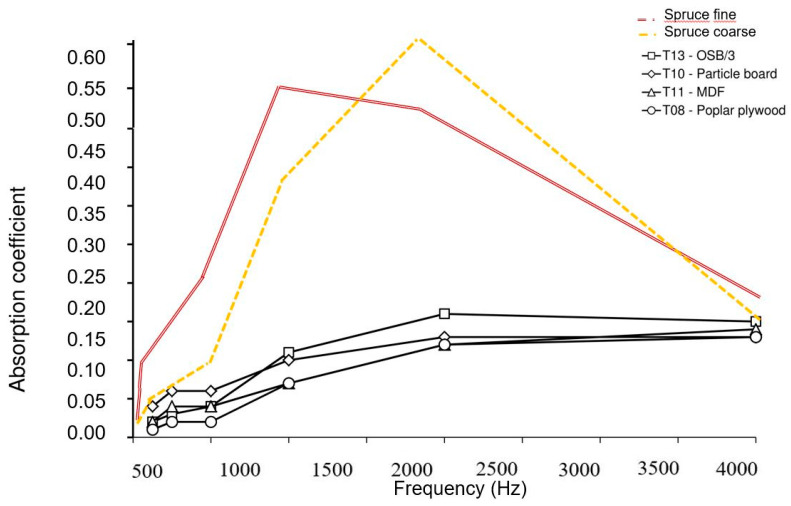
Dependence of the sound absorption coefficient on frequency for wood-based composites with a thickness 16 < x < 18.4 mm and a density 500 < y < 600 kg/m³ (after Smardzewski et al. [9]).

In the case of medium thicknesses (16 < x < 18.4mm), the wood-based materials with densities from 481 kg/m³ (T10, particleboard), 515 kg/m³ (T08, poplar plywood), 558 kg/m³ (T11, medium density fibreboard, MDF) and 613 kg/m³ (T13, oriented strand board) did not reach sound absorption coefficients higher than 0.15 (Figure 5). The spruce bark samples with similar density levels (414 kg/m³ for spruce coarse and 500 kg/m³ for spruce fine) can absorb sound better at frequencies between 250 and 4000 Hz, with two peaks at 0.52 (spuce fine, at 1000 Hz) and 0.61 (spruce coarse, at 2000 Hz). 

**Figure 6:**
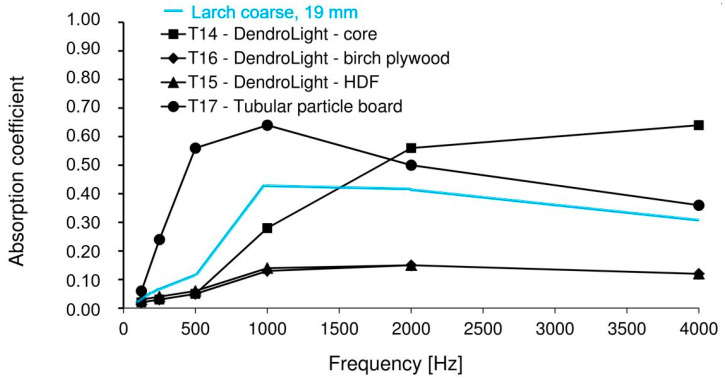
Dependence of the sound absorption coefficient on frequency for wood-based composites with a thickness 22.3 < x < 28 mm and a density 220 < y < 500 kg/m³ (after Smardzewski et al. [9]).

For thickness interval 22.3 < x < 28 mm, the wood-based materials with densities that raged from 220 kg/m³ (T17, tubular particle board), 270 kg/m³ (T14, DendroLight—core), 459 kg/m³ (T15, DendroLight—planked with HDF) and 493 kg/m³ (T16, DendroLight—planked with birch plywood) performed better to the larch bark board with a thickness of 19.3 mm (Figure 5). The best values of α coefficient (0.06, 0.24, 0.56, 0.64, 0.5 and 0.36) were recorded for the T17 panels, with the best acoustic absorbability for all frequencies for the interval between 125 and 4000 Hz. At 1 kHz the 19 mm larch bark panel recorded an α coefficient with 35% smaller than the T17 tubular particleboard, and at 2 KHz was 20% reduced, but these results are consistent with the data shown in Figure 6. These outputs correspond with experimental data of Karlinasari et al. [63], Yang et al. [64], Zulkifli et al. [65,66] and Smardzewski et al. [67], where the maximum sound absorption coefficient was achieved by wood and wood composites at middle frequencies (about 1500–3000 Hz). These results are also in compliance to authors [9,14,68,69,70], in that the best sound absorption capabilities of wood based composites were achieved by the samples with low density of surface layers and high porosity. 

The human ear is most sensitive to noise at central frequencies [71], therefore bark-based insulation panels can be recommended as sound insulation applications.

## 4. Conclusions

This paper reveals that the sound insulation properties of the tested larch and spruce bark panels open a new subject related to the advantages of the use of wood biomass. The thickness of the board plays an important role for the sound absorption coefficient.

The sound absorption coefficient for the panel with a thickness of 19 mm were compared with wood-based composites with similar thicknesses and it was found that the spruce bark panels (with coarse and fine particles) can absorb sound even better than OSB, particleboard, MDF and poplar plywood, therefore the values of the sound absorption coefficient confine their application as structural elements for reducing the noise effects in residential buildings, and open new ways for a deeper research in this field. 

The spruce composite panels with densities lower than 500 kg/m³ are able to attenuate up to 60% more sound, compared to densified larch agglomerated boards (600, respectively 700 kg/m³).

In case of using a clearance wall (Figure 3), the sound absorption effect is high (0.7 for the panels with spruce with fine-grained particles and with larch bark) at low frequencies, starting from 125 Hz. These findings encourage further investigations about sound insulation properties of the bark-based insulation panels.

This research brings an expanding knowledge in the area of composites based on softwood bark and emphasizes on the advantages of an underrated material, namely tree bark, that can substitute wood and other materials that involve more grey energy that affect their life cycle assessment.

## Figures and Tables

**Table 1:** Particle size, dimensions, density levels and adhesive type of the bark insulation boards.

Board Type	Bark Particle Size (mm)	Board Thickness (mm)	Boards Dimension (mm)	Board Density (kg/m³)	Adhesive
Spruce fine	8–13	21	500 × 500	500	10% UF
Spruce coarse	10–30	20	500 × 500	414	10% UF
Larch coarse, thin	10–30	11	500 × 500	690	10% tannin
Larch coarse, thick	10–30	19	500 × 500	571	10% tannin
